# Clinical Observation of Posterior-Chamber Phakic Implantable Collamer Lens V4c Implantation in Myopic Patients with Shallow Anterior Chamber Depth: A Retrospective, Consecutive Observational Study

**DOI:** 10.1155/2024/3181569

**Published:** 2024-05-22

**Authors:** Juan Yuan, Shuang Wu, Zongli Hu, Chunlin Chen, Shiyang Ye, Jian Ye

**Affiliations:** ^1^Department of Ophthalmology, Daping Hospital, Army Medical Center of PLA, Army Medical University, Chongqing, China; ^2^Branch of Chongqing Clinical Research Center for Eye Diseases, Chongqing, China; ^3^Department of Ophthalmology, Southwest Hospital, Army Medical University, Chongqing, China

## Abstract

**Purpose:**

The reference range for the preoperative anterior chamber angle width for ICL surgery is unclear. Our objective was to assess the clinical effect and the range of anterior chamber angle width of posterior-chamber implantable collamer lens V4c (ICL V4c) implantation in patients with anterior chamber depth (ACD) < 2.8 mm.

**Methods:**

Patients who underwent ICL V4c implantation with shallow ACD were included in this retrospective study. The patients' uncorrected and corrected distance visual acuity, angle of trabecular-iris (TIA), angle-opening distance (AOD500), trabecular-iris space area (TISA500), corneal endothelial cell density, vault, retinal nerve fiber layer thickness, intraocular pressure, visual field, and complications were analyzed.

**Results:**

Forty-one patients (68 eyes) completed at least 12 months of follow-up (median follow-up, 30 months). The effectiveness and safety indices were 1.09 ± 0.13 and 1.04 ± 0.21, respectively. The preoperative TIA values on the nasal and temporal sides were 39.78 ± 7.68 degree (range, 25.8-65.1 degree) and 41.54 ± 8.03 degree (range, 28.5-63.00 degree). Forty-seven eyes had uncorrected distance visual acuity ≥1.0, and 55 had corrected distance visual acuity ≥1.0 at the last follow-up visit. The TIA, AOD500, and TISA500 on the nasal and temporal sides were significantly reduced compared to those before surgery (all *P* < 0.01); no eye had an angle closure or elevated intraocular pressure. The ICL V4c vault was 290.88 ± 153.36 *μ*m (range, 60.0-880.0 *μ*m). No severe complications occurred in any patient.

**Conclusions:**

In patients with myopia with shallow ACD (2.55-2.79 mm), a preoperative TIA >25.8° is safe and effective for a relatively long time after surgery; however, an extended long-term close follow-up is needed.

## 1. Introduction

Myopia has become a global public health problem. The novel coronavirus disease epidemic has accelerated the increase in myopia cases [1], and an increasing number of people are eager to get rid of their glasses. The main surgical options for myopia correction include corneal laser and intraocular refractive surgery. For patients with special conditions, such as high myopia, thin corneas, or suspicious topography, who are not eligible for corneal laser surgery but have certain occupational needs and a strong desire to remove their lenses, posterior-chamber phakic implantable collamer lens (ICL V4c) implantation is a better option. The Visian implantable collamer lens V4c with a hole (STAAR Surgical Inc, Monrovia, USA) is the most popular and widespread phakic intraocular lens type [2, 3]. Individuals with anterior chamber depth (ACD) < 2.8 mm are generally not recommended for ICL surgery because the low vault may be associated with shallow ACD and elevated intraocular pressure (IOP) after surgery, and such patients are more likely to develop anterior subcapsular cataracts [4–6]. The postoperative anterior chamber angle width was significantly reduced with this procedure [7–9]. Qian et al. studied patients with shallow ACD and found that the anterior chamber angle width was narrower in patients with elevated IOP after ICL surgery for at least 24 months [10]. However, Kamiya et al. reported that narrowing the anterior chamber angle width did not significantly affect endothelial cell density (ECD) or IOP in ICL implanted eyes during the 18-month postoperative period [11]. There is no clear reference range regarding the preoperative anterior chamber angle width. This study aimed to assess clinical results and the range of anterior chamber angle width of the implantation of the ICL V4c in patients with shallow ACD (<2.8 mm) for at least 12 months.

## 2. Materials and Methods

### 2.1. Study Design and Population

This was a retrospective, observational study of consecutive myopic patients who had an EVO-ICL implantation from May 2018 to June 2021 at Daping Hospital's Department of Ophthalmology, Army Medical University. Before surgery, each patient was informed of the risks and possible outcomes of the surgery and signed an informed consent. The protocol of this study complied with the Declaration of Helsinki and received approval from the Daping Hospital's Ethics Committee, Chongqing, China (Ethical approval number: 2022-196).

The inclusion criteria were: (1) age 18–40 years before surgery, (2) stable refraction error (an increase of ≤0.50 diopters [D] in the previous 2 years), (3) ACD <2.80 mm, and (4) a strong desire to discontinue the use of glasses or contact lenses and the ability to withstand the risks of the procedure. The exclusion criteria were: (1) endothelial cell density <2,000 cells/mm^2^, (2) a history of any ocular disease or surgery (e.g., corneal disease, uveitis, and glaucoma), and (3) uncontrolled systemic disease (e.g., diabetes and hyperthyroidism).

### 2.2. Examinations

The patients underwent routine preoperative examinations, including uncorrected and corrected distance visual acuity (UDVA and CDVA recorded in decimals), funduscopic examinations, slit-lamp biomicroscopy, and assessments of the manifest refraction, corneal ECD (EM-3000, Tomey, Japan), IOP (TX-20, Canon, Japan), ACD, white-to-white diameter (Pentacam, Oculus Inc., Wetzlar, Germany), axial length, and pupil diameter (IOL Master500, Carl Zeiss Meditec).

**Figure 1:**
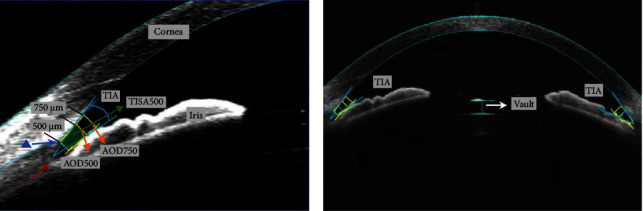
Measurement of the anterior chamber angle opening parameters using anterior-segment optical coherence tomography (Visante OCT, Carl Zeiss Meditec). (a) The angle opening distance (AOD) is the perpendicular distance from the iris to the trabecular meshwork, and can be measured at different distances anterior to the scleral spur, such as 500 and 750 *μ*m (AOD500 and AOD750, respectively). AOD500 was defined as the perpendicular distance between the point on the trabecular network at 500 *μ*m from the scleral spur and anterior iris surface. The TIA (angle of trabecular-iris) was defined as the angle between the following two lines: a line from the apex of the iris recess to the AOD500 point on the corneal surface, and another line from the AOD500 point on the iris surface to the apex of the iris recess. TISA500 was defined as the surface area of a trapezoidal region consisting of four lines: AOD500, a line drawn from the scleral spur perpendicular to the trabecular meshwork toward the iris surface, the inner corneoscleral wall, and the anterior surface of the iris. (b) The central vault was defined as the perpendicular distance from the posterior surface of the ICL to the anterior surface of the crystalline lens. △ = scleral spur, ^*∗*^ = the apex of the iris recess.

The anterior chamber angle opening parameters (angle of trabecular-iris (TIA), angle-opening distance (AOD500), and trabecular-iris space area (TISA500)), preoperative ACD, and postoperative vault with anterior-segment optical coherence tomography (Visante OCT, Carl Zeiss Meditec) are presented in Figure 1. The OCT imaging was obtained from a single horizontal scan (temporal-nasal) under mesopic conditions (500 lux) with constant artificial lighting. All patients were assessed in their natural state (without pharmacological miosis and mydriasis) by the same physician within consistent room conditions. ACD was defined as the distance between the center of the corneal endothelium to the crystalline lens. The average thickness of retinal nerve fiber layer (RNFL) (Cirrus HD-OCT, Carl Zeiss Meditec, Dublin, CA, USA) and visual fields (Humphrey Field Analyzer, Carl Zeiss Meditec) was evaluated. After surgery, the patients were followed up according to the prescribed schedule (1 day; 1 week; 1, 3, 6, and 12 months; and then annually).

### 2.3. Surgical Procedure

A senior and experienced surgeon performed all procedures. Preoperative horizontal axis markings at the seated level were performed in patients implanted with a TICL. Following topical anesthesia and mydriasis, a clear corneal incision was made at the temporal limbal. The ICL/TICL was implanted with a special injector cartridge via the tunnel incision after 1% sodium hyaluronate was injected into the anterior chamber, and its four haptics were adjusted to the ciliary sulcus. Subsequently, a balanced salt solution was used to remove the hyaluronate completely. After surgery, levofloxacin was administered for 3 days, and the following were prescribed: sodium hyaluronate eye drops (artificial tears) for 1 month, nonsteroidal anti-inflammatory eye drops (0.1% bromfenac ophthalmic eye drops) for 20 days, and steroidal eye drops (Lotemax suspended eye drops) for 7 days.

### 2.4. Statistical Analysis

SPSS 26.0 software (SPSS Inc., IBM Corporation) was used for statistical testing. Data were reported as mean ± standard deviation (SD) or median (P25, P75) (range, minimum  - maximum). The Kolmogorov–Smirnov tests were used to confirm normality. Data were tested using paired-samples *t*-tests or Wilcoxon signed-rank test based on normality or nonnormality. Visual acuity was converted to Logmar criteria for statistical analysis. The Pearson or Spearman rank correlation was used to assess correlations between the TIA, ACD, spherical equivalent (SE), vault, IOP, axial length, and average RNFL thickness as needed. *P* values <0.05 were considered statistically significant.

## 3. Results

### 3.1. Patient Demographics

We screened 88 patients during this period, but only 41 completed the required follow-up. Overall, 68 eyes of 41 patients (median age, 27 years) were included. Toric and nontoric ICL models were implanted in 40 (58.8%) and 28 (41.2%) eyes, respectively, and observed for at least 12 months, with a median follow-up of 30 months and a maximum follow-up of 46 months. The patients' preoperative characteristics are summarized in Table 1.

### 3.2. Safety and Effectiveness

**Figure 2:**
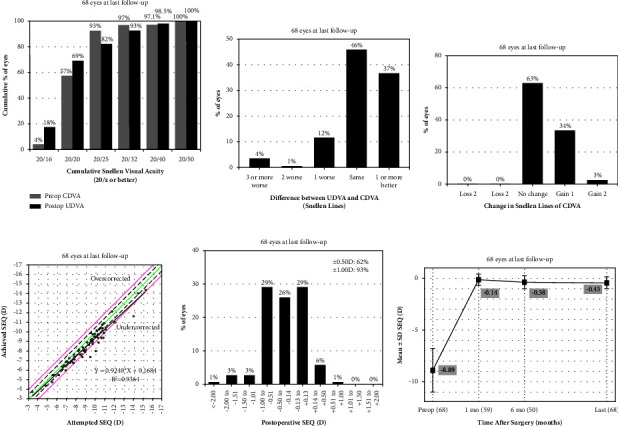
(a) Comparison of postoperative uncorrected distance visual acuity (UDVA) at the last follow-up and preoperative corrected distance visual acuity (CDVA); (b): difference between postoperative UDVA and preoperative CDVA; (c) change in Snellen lines of CDVA at last follow-up visit; (d) attempted spherical equivalent refraction change versus the achieved spherical equivalent refraction change; (e) distribution of spherical equivalent refractive accuracy; (f) stability of spherical equivalent refraction. UDVA, uncorrected distance visual acuity; CDVA, corrected distance visual acuity; D, dioptre; VA, visual acuity; SEQ, spherical equivalent. Postop, postoperative; preop, preoperative; mo, months.

At the last follow-up, the median logMAR of CDVA was 0.00 (0.00, 0.00) (range, −0.08-0.4), and the median logMAR of UDVA was 0.00 (0.00, 0.10) (range, −0.1-0.4). The safety index (mean postoperative CDVA/mean preoperative CDVA) was 1.09 ± 0.13 (range, 1.0-1.5), and the efficacy index (mean postoperative UDVA/mean preoperative CDVA) was 1.04 ± 0.21 (range, 0.5-1.5). Overall, 47 eyes (69.1%) had a postoperative UCVA 20/20 or better, 68 eyes (100%) achieved postoperative UDVA 20/50 or better (Figure 2(a)). Fifty-six (82.4%) had a postoperative UCVA consistent with or better than the preoperative CDVA (Figure 2(b)). No patient had CDVA loss at the final follow-up; 23 eyes (33.8%) gained one line, and 2 (3.0%) gained two lines (Figure 2(c)).

### 3.3. Predictability and Stability

A scatter plot of the attempted versus achieved SE corrections and the distribution of postoperative SE refraction accuracy are shown in Figure 2(d) and 2(e), respectively. At the last visit, 35 eyes (51.5%) were within ±0.5 D of the attempted SE. Sixty eyes (88.2%) had a postoperative SE within ±1.0 D of the attempted SE (Figure 2(d)). Forty-two eyes (61.8%) achieved a residual SE within ±0.5 D, and 63 (92.6%) achieved a residual SE within ±1.0 D (Figure 2(e)). The average SE were −0.14 ± 0.54 D and −0.43 ± 0.55 D at 1 month and last follow-up, respectively, and overall myopia progressed −0.35 ± 0.49 D (*P* < 0.01) (Figure 2(f)).

### 3.4. IOP, Corneal ECD, and Vault

The IOP range was 9.3-22.0 mmHg (15.79 ± 2.80 mmHg) at the last follow-up, with two eyes ≥21 mmHg (21 mmHg in one eye and 22 mmHg in the other), and the difference in IOP before and after surgery shows no statistical significance (*P*=0.294). Two eyes had a preoperative IOP of 22 mmHg, and the diagnosis of glaucoma was ruled out after a thorough examination. Postoperatively, the IOP in the two eyes was 22 mmHg, and the nasal and temporal TIA were 31.1 and 33.1° and 21.9 and 25.1°, respectively. We examined the optic nerve, optic disc, and visual field in detail and found no signs of glaucoma; however, subsequent long-term observation is necessary. We found no significant association between the preoperative ACD and the postoperative IOP (Spearman correlation analysis: *r* = −0.075, *P*=0.543). The ECD was 2627.88 ± 257.48 cells/mm^2^ (range, 2,151-3,326 cells/mm^2^); the mean loss rate was 0.79 ± 7.46% compared to the preoperative ECD, with no significant difference (*P*=0.334). The mean vault at 1 week and the last examination was 442.50 ± 192.14 *μ*m (range, 80-1140) and 290.88 ± 153.36 *μ*m (range, 60-880 *μ*m), respectively. Overall, the vaults of 37 eyes (54.4%) were between 250 and 750 *μ*m, and those of 26 eyes (38.2%) were between 100 and 250 *μ*m. One eye had a vault of 880 *μ*m and a nasal TIA of 8.3°. In four eyes (5.9%), the vault was <100 *μ*m: 60 *μ*m in two eyes, 80 *μ*m in one, and 90 *μ*m in the other. In our examination of all four eyes, the anterior chamber was stable and clear, with no lens opacity observed.  Table 2 summarizes the main ocular parameters for the five eyes with low and high vaults.

### 3.5. Angle Opening Parameters

**Figure 3:**
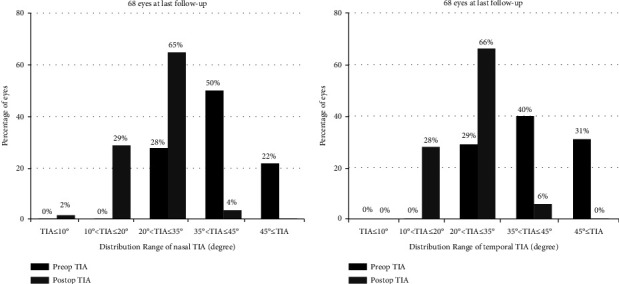
Comparison of the last follow-up and preoperative TIA on nasal (a) and temporal (b). TIA: angle of trabecular-iris.

**Figure 4:**
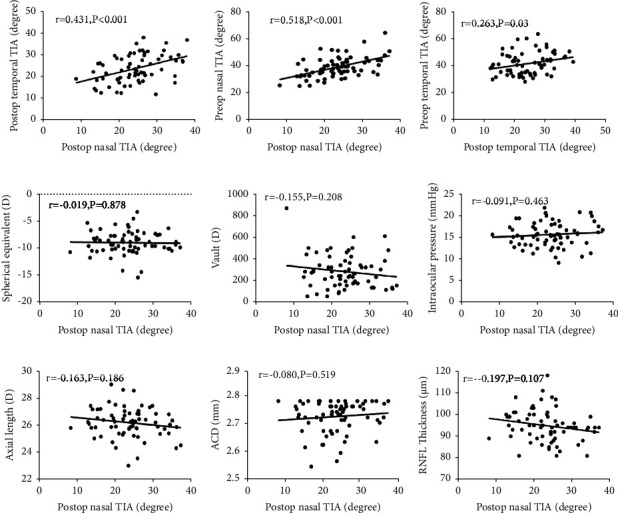
Scatter plot of the correlation between the postop TIA and other ocular parameters. (a) Postop nasal TIA and postop temporal TIA. (b) Postop nasal TIA and preop temporal TIA. (c) Postop temporal TIA and preop temporal TIA. (d) Postop nasal TIA and spherical equivalent (SE). (e) Postop nasal TIA and vault. (f) Postop nasal TIA and intraocular pressure (IOP). (g) Postop nasal TIA and axial length. (h) Postop nasal TIA and ACD. (i) Postop nasal TIA and RNFL thickness. Postop, postoperative; preop, preoperative; TIA: angle of trabecular-iris; ACD: anterior chamber depth; RNFL thickness: retinal nerve fiber layer thickness.

Before surgery, the temporal side angle opening parameters (TIA, AOD500, and TISA500) were slightly larger than those of the nasal side (*P* < 0.05, paired *t*-test), with a significant decrease in the angle opening parameters at the last visit (*P* < 0.001, paired *t*-test). The TIA decreased significantly on the nasal and temporal sides at the last follow-up by 41.5% and 41.3%, respectively, compared to the preoperative values (from 39.78 ± 7.68° [range, 25.8°-65.1°] and 41.54 ± 8.03° [range, 28.5°-63.0°] to 23.47 ± 6.49° [range, 8.3°-37.1°] and 24.38 ± 6.61° [range, 12.2°-39.2°], respectively). Preoperatively, one eye (2%) had a minimum nasal TIA of 25.8° and a postoperative nasal TIA of 13.5°, with a clear and stable anterior chamber. At the last follow-up, one eye (2%) had a nasal TIA of 8.3° (Table 2), 20 eyes (29%) had a nasal TIA between 10° and 20°, and 19 eyes (28%) had a temporal TIA between 10° and 20°. The distribution of the nasal and temporal TIA values is shown in Figure 3(a) and 3(b). No statistically significant difference was observed between the nasal and temporal TIA at the last follow-up. We analyzed the correlation of nasal TIA at the final follow-up with the preoperative nasal TIA, preoperative SE, axial length, vault, final IOP, and RNFL thickness (Figure 4). There was a correlation between the nasal and temporal TIA at the last visit (Spearman correlation analysis: *r* = 0.431, *P* < 0.001; Figure 4(a)). The final TIA was significantly correlated with the preoperative nasal and temporal TIA (Spearman correlation analysis: *r* = 0.518, *P* < 0.001; *r* = 0.263, *P*=0.03; Figure 4(b) and 4(c)). We found no significant correlation between the TIA values at the last examination and the preoperative SE, vault, final IOP, axial length, preoperative ACD, and RNFL thickness (Figure 4(d) and 4(i)). In addition, the ICL vault showed a weak correlation with preoperative TIA values (Pearson correlation analysis: nasal TIA *r* = −0.344, *P* < 0.05 and temporal TIA *r* = −0.334, *P* < 0.05, respectively).

At the last follow-up, the AOD500 was significantly reduced on the nasal and temporal sides by 50.2% and 48.1%, respectively, compared to the preoperative values (from median 0.490 [0.411, 0.611] mm and median 0.505 [0.404, 0.670] mm to 0.244 ± 0.078 mm and 0.262 ± 0.094 mm, respectively). The TISA500 was significantly reduced on the nasal and temporal sides by 47.5% and 46.8%, respectively, compared to the preoperative values (from median 0.160 mm^2^ [0.130, 0.225] mm^2^ and median 0.171 [0.133, 0.235] mm^2^ to median 0.084 [0.061, 0.093] mm^2^ and 0.091 ± 0.037 mm^2^, respectively). Tables 3 and 4 present a comparison of the angle opening parameters.

### 3.6. Mean RNFL Thickness and Visual Field

At the last follow-up, the mean RNFL thickness increased from its preoperative value of 93.06 ± 6.67 *μ*m to 94.88 ± 7.41 *μ*m, showing a statistically significant increase of 2.0% (*P* < 0.01). All patients underwent visual field examination, and no glaucomatous visual field manifestations, such as paracentral scotomas, arcuate scotoma, or nasal step, were found.

During the follow-up, there was no anterior subcapsular opacity or secondary glaucoma and no eye required secondary surgery.

## 4. Discussion

Since the US Food and Drug Administration approved the Visian ICL (STAAR Surgical) in 2005, the procedure has been widely used and is safe, effective, and reversible in the correction of moderate to high myopia [12–15]. It is possible to preserve the lens and adjustment function of the cornea after ICL implantation in young patients without altering the cornea's biological structure [16]. Patients with low-to-moderate myopia can also achieve satisfactory results after ICL surgery [17, 18]. ICL has been used in Europe since 1997, and manufacturers recommend ACD >2.8 mm for myopia and 3.0 mm for hypermetropia [2]. The most common and worrisome adverse events after ICL implantation are related to the vault [14]. An excessively high vault may cause the ICL to push the iris forward and cause the angle to become narrow or close, causing corneal endothelial damage, severe pupillary block, or even secondary glaucoma [19]. If the vault is too low, it may lead to the opacity of the lens (model V4 ICL) or rotation of the TICL due to instability and affect corrected vision [20–22]. The IOP is also crucial after ICL implantation. The IOP is negatively correlated with the preoperative ACD but still within the normal range, as reported by Vanathi et al. [23]. In our study, there was no significant correlation between preoperative ACD and postoperative IOP, probably because of the narrow range of our ACD. The ICL V4c is designed to reduce the impact on aqueous humor (with a 360 *μ*m central hole), which significantly reduces the risk of acute IOP elevation and the incidence of cataracts [24, 25].

Chinese individuals have a lower ACD than Caucasians, according to glaucoma-related studies [26], and the ACD is a major determinant of angle width [27]. Therefore, preoperative evaluation of all ocular biological parameters is crucial in patients with myopia with an ACD <2.8 mm in China. The present study reported on the effectiveness and safety and assessed the extent of the anterior chamber angle width in patients with shallow ACD (<2.8 mm) who have been implanted with ICL V4c for over a year.

We observed patients with a preoperative ACD <2.8 mm (range, 2.55-2.79 mm). These patients had a wide range of preoperative SE (range, −15.2-3.25°), with a median follow-up of 30 months (range, 12-46 months). The effectiveness and safety indices were 1.0, similar to that in Lisa et al.'s [28] 1-year observation of patients with an ACD ≥2.8 mm, demonstrating good safety and efficacy. Niu et al. [29] also studied patients with shallow ACD (<2.8 mm) and observed satisfactory and stable visual results 1 year after surgery.

We used anterior-segment optical coherence tomography (AS-OCT) to observe the anterior chamber angle width and vault, which has a higher reproducibility of imaging than conventional anterior chamber angioscopy and can be used as a documentation tool for long-term follow-up. Previous studies revealed the anterior chamber structure and vault change with different pupil diameters, with larger pupil sizes resulting in a higher vault [30–33]. Gonzalez-Lopez et al. found that in pupillary miosis, the iris pushes the lens downward, accompanied by a widening of the anterior chamber angle and a lowering of the vault [31]. Preoperative and postoperative AS-OCT measurements were performed under identical external conditions (ambient light 500 lux) for better longitudinal comparisons. ICL implantation results in significant narrowing of the TIA in patients with preoperative ACD ≥2.8 mm [7–9, 11]. In the present study, angle opening parameters (TIA, AOD500, and TISA500) were significantly reduced at the final follow-up. The nasal and temporal TIA values at the last follow-up were reduced by 41% compared to the preoperative values. A previous study reported a 40.4% reduction in the TIA from the preoperative value (38.1 ± 9.7° to 22.7 ± 5.9°) 1 year after ICL implantation using UBM measurements, with no significant reduction in the following 2 years [8]. Fernández-Vigo et al. [7] observed patients with ACD ≥2.8 mm measured by OCT and observed significantly reduced TIA values within 1 month after surgery, which remained stable after 2 years. In the present study, although the patients' ACD was <2.8 mm before surgery, the rate of reduction of the TIA at >12 months postoperatively was 41%. The postoperative TIA did not decrease more than that of patients with ACD ≥2.8 mm, consistent with the findings of Chung et al. [8] and Fernández-Vigo et al. [7].

In this study, patients had a wide preoperative TIA, with minimum and maximum TIA values of 25.8° and 65.1°, respectively. The patient with a preoperative nasal TIA of 25.8° had a nasal TIA of 13.5° and vault of 450 *μ*m at the 32-month postoperative follow-up. According to the Shaffer angle grading (the angle between two hypothetical tangents on the inner side of the cornea and the anterior side of the iris recess), an angle width <20° is at risk of angle closure, whereas an extremely narrow angle width ≤10° is highly likely to close. Qian et al. also studied eyes with an ACD <2.8 mm and found that those with elevated postoperative IOP had a lower anterior chamber angle and higher vault. Preoperatively, the TIA was >20° in all eyes measured using AS-OCT; one eye (2%) had a nasal TIA <10° at the last follow-up, with a vault of 880 *μ*m and preoperative nasal TIA of 26.2°. The anterior chamber was clear and stable, the temporal TIA was 19.6°, and the IOP was <21 mmHg. The patient's angle width and IOP will be closely monitored during subsequent follow-up, and ICL replacement or repositioning will be performed if necessary. In Fernández-Vigo et al.'s study [7], 14.8% of the eyes showed iris trabecular contact, but no progression was detected within 2 years; because of the remaining open angles in the other quadrants, an increase in IOP did not occur. Clinicians must assess the anterior chamber angle width and shallow ACD before selecting the appropriate ICL size to ensure a successful ICL surgery. Before surgery, the lowest ACD was 2.55 mm in one eye whose preoperative nasal and temporal TIA values were 35.7° and 35.5°, respectively; those values were reduced to 16.9° and 15.6°, respectively, at the 17-month follow-up, with a postoperative vault of 350 *μ*m. Although the ACD is minimal, the postoperative result is noteworthy. Lim et al. [6] reported that patients with an ACD <2.8 mm had narrower postoperative angle opening parameters than those with an ACD ≥2.8 mm, although all were within the acceptable range. In a study on glaucoma, Xu et al. [27] found that the ACD was the strongest determinant of angle width. In the present study, no significant correlation between the TIA and ACD was found, probably because of the small range of the ACD (2.55-2.79 mm) and the small number of patients. The sample size should be expanded in subsequent studies to include patients with a more extensive range of ACD (≥2.8 mm).

Currently, the more desirable vault range is 250-750 *μ*m, equivalent to a corneal thickness of 0.5-1.5. Previous research has established that the vault will be reduced with light-induced pupil reduction [30–32, 34]. Gonzalez-Lopez et al. have demonstrated that the vault changes dynamically rather than being fixed; they also defined the vault range (the amount of vault change measured at the time of the maximum light-induced pupil change) as a parameter describing the dynamics of vault change [31]. The light-induced changes in pupil dynamics after ICL implantation were further evaluated in their subsequent study, which found that the vault was positively correlated with pupil diameter changes at different luminosities. Under photopic light conditions (990 lux), the mean value of the vault was 412 ± 177 *μ*m (range, 76-845 *μ*m), and under maximum mydriasis (0.5 lux), it was 506 ± 190 *μ*m (range, 122-903 *μ*m); when the vault range was 95 ± 51 *μ*m (range, 13-277 *μ*m), the pupil diameter changed from 3.48 ± 0.61 mm to 5.84 ± 0.77 mm [32]. This indicates that the vault increases and decreases as the pupil dilates or constricts. Our data also support the view that the vault is dynamic. At the last follow-up, the vault was <100 *μ*m measured using AS-OCT in four eyes under light conditions (artificial light, 500 lux), with a corneal thickness of approximately 0.2 CT under a slit lamp. Under scotopic conditions (0 lux), the vault had a corneal thickness of approximately 0.3 under a slit lamp biomicroscope, with a clear and stable anterior chamber and no anterior subcapsular opacity. In our study of patients with shallow anterior chambers and low vault, there was also an increase in vault (approximately 0.1 corneal thickness) in the darkroom (0 lux) compared to that with room luminance (500 lux); however, the amount of change is different from that of the previous study [32] due to differences in luminosity and the measurement method. Rayner et al. [35] reported that a vault ≥50 *μ*m can be considered a safe level without an upper limit as long as the angle structure and function are normal. In a study by Gonzalez-Lopez et al. [36] on patients with low vault (vault in photopically induced miosis <100 *μ*m), a prolonged low vault did not increase the risk of cataract development but required safe observation without initial exchange. Chen et al. [37] demonstrated that preoperative ACD and central vault were positively correlated, possibly because the preoperative ACD was related to the ICL size. However, all patients in the present study had shallow ACD. Lim et al. [6] also evaluated patients with shallow preoperative ACD and found a postoperative vault lower than expected, with a potential risk of cataract formation (model V4 without central hole). At the last follow-up, we found a weak correlation between the vault and nasal and temporal TIA values, consistent with the findings of Eissa et al. [38]. No anterior subcapsular cataract was found, but a 20 *μ*m increase in the anterior surface of the crystalline per year with age will result in an approximately 28 *μ*m yearly decrease in the vault [39]. Therefore, it is essential to follow up with patients regularly for a long time after surgery and to avoid the influence of pupil size on the measurement results.

At the last visit, no significant elevations were found in the IOP; the mean RNFL thickness was slightly higher than the preoperative value. Cheng et al. [40] studied patients aged 18 to 40 years and found that patients with myopia with axial length >26 mm had thicker mean and temporal RNFL than those with axial length <26 mm (119 ± 17.4 *μ*m, 97.75 ± 30.36 *μ*m vs. 105.85 ± 12.83 *μ*m, 67.70 ± 9.61 *μ*m). However, this change requires further investigation. Patients with early visual field changes in glaucoma were also not identified. Our mean ECD loss rate was 0.79 ± 7.46%, with no significant difference in endothelial cells compared to the preoperative values. Niu et al. [29] observed patients whose preoperative ACD was <2.8 mm at >1 year after ICL implantation and found that the ECD loss was 8.38%. We have a low loss of ECD, and ECD loss was associated with intraoperative manipulation, postoperative inflammation, and physiological loss, suggesting that ICL V4c implantation had a minimal impact on the corneal endothelium. However, long-term observations of endothelial cell counts are required.

The present study had some limitations. First, the retrospective methodology is based on a single institution, which may lead to missing data for different periods and the exclusion of patients with good postoperative outcomes who could not revisit the doctors. Moreover, we did not measure TIA in the superior and inferior quadrants. Finally, due to the unique nature of patients with shallow anterior chambers, the surgeon's thorough consideration of surgical risks and preoperative examinations, and the patient's tolerance, the sample size is limited. It is important to note that dynamic observation of the vault is a more comprehensive assessment of the safety of the procedure. Patients with a larger range ACD (>2.8 mm) could be evaluated for a longer period using a prospective comparative analysis. This will be informative in evaluating preoperative anterior-segment biologic parameters, including ACD and anterior chamber angle in patients undergoing ICL surgery.

## 5. Conclusions

In patients with myopia shallow ACD (<2.8 mm), significant narrowing of the anterior chamber angle width occurred after ICL implantation. Nevertheless, patients with a preoperative TIA >25.8° showed good results for a certain period after surgery. All patients had no angle closure, anterior subcapsular opacity, or secondary glaucoma complications after more than 12 months of surgery; however, regular long-term follow-up is still necessary.

## Figures and Tables

**Table 1:** Distribution of preoperative characteristics (mean ± SD and *M* (P25, P75)).

Parameters	Preoperative values		Range (min, max)
	Mean ± SD	*M* (P25, P75)	
*N*, eyes	68		
Age (years)	27.21 ± 6.11	27.00 (22.25, 29.75)	18, 40
Spherical equivalent (D)	−8.89 ± 2.11	−9.00 (−10.09, −7.53)	−15.25, −3.25
Spherical (D)	−8.19 ± 1.97	−8.25 (−9.25, −7.00)	−14.25, −3.00
Cylindrical (D)	−1.40 ± 1.49	−1.0 (−1.75, −0.50)	−7.25, 0
CDVA (LogMAR)	0.05 ± 0.09	0.00 (0.00, 0.10)	−0.08, 0.40
ACD (mm)	2.73 ± 0.06	2.75 (2.71, 2.78)	2.55, 2.79
IOP (mmHg)	16.11 ± 2.42	16.25 (14.63, 17.50)	9.9, 22.0
ECD (cells/mm^2^)	2650.63 ± 194.88	2654.00 (2516.50, 2788.00)	2267.00, 3211.00
ICL size (mm)	12.52 ± 0.35	12.6 (12.1, 12.6)	12.1, 13.2
Axial length (mm)	26.23 ± 1.09	26.26 (25.77, 26.90)	23.05, 29.06
WTW (mm)	11.25 ± 0.35	11.25 (11.00, 11.50)	10.65, 12.30

D, diopters, CDVA, corrected distance visual acuity; LogMAR, logarithm of the minimal angle of resolution; IOP, intraocular pressure; ACD, anterior chamber depth; ECD, corneal endothelial cell density; ICL, implantable collamer lens; WTW,white-to-white; 1 mmHg = 0.133 kPa.

**Table 2:** Cases with vault less than 100 *μ*m and more than 750 *μ*m.

	Follow-up (month)	Age (year)	Preoperative					Postoperative	
			SE (D)	ACD (mm)	TIA (N, T, °)	WTW (mm)	ICL size	TIA (N, T, °)	Vault (*μ*m)
Case 1	42	29	−6.63	2.74	32.8, 34.8	11.2	12.6	13.7, 18.9	60
Case 2	32	37	−7.63	2.78	45.1, 40.9	12.3	13.2	18.9, 13.1	60
Case 3	15	25	−6.38	2.71	26.0, 32.5	11.6	12.6	16.2, 21.7	80
Case 4	30	21	−8.36	2.77	36.7, 34.4	10.9	12.1	21.9, 25.1	90
Case 5	32	31	−10.63	2.79	26.2, 32.4	11.3	12.6	8.3, 19.6	880

ACD, anterior chamber depth; WTW, white-to-white; N, nasal side; T, temporal side. TIA (trabecular-iris angle at 500 *μ*m) was defined as a line from the apex of the iris recess to the AOD500 *μ*m point on the corneal surface, and then another line from the AOD500 *μ*m point on the iris surface to the apex of the iris recess, the angle between the two lines. Case with postoperative TIA less than 10 degrees.

**Table 3:** Comparison of anterior chamber angle opening parameters at the nasal side before and after ICL implantation.

Time	*N*	TIA (degree)	AOD500 (mm)	TISA500 (mm^2^)
Preoperative	68	39.78 ± 7.68	0.490 (0.411, 0.611)	0.160 (0.130, 0.225)
Range (min, max)		(25.8, 65.1)	(0.238, 1.064)	(0.060, 0.361)
Postoperative	68	23.471 ± 6.49	0.244 ± 0.078	0.084 (0.061, 0.093)
Range (min, max)		(8.3, 37.1)	(0.092, 0.484)	(0.034, 0.198)
*t*/*z*		19.11	−7.168	−7.115
*P*		<0.001	<0.001	<0.001

**Table 4:** Comparison of anterior chamber angle opening parameters at temporal side before and after ICL implantation.

Time	*N*	TIA (degree)	AOD500 (mm)	TISA500 (mm^2^)
Preoperative	68	41.54 ± 8.03	0.505 (0.404, 0.670)	0.171 (0.133, 0.235)
Range (min, max)		(28.5, 63.00)	(0.274, 1.096)	(0.081, 0.392)
Postoperative	68	24.38 ± 6.61	0.262 ± 0.094	0.091 ± 0.037
Range (min, max)		(12.2, 39.2)	(0.105, 0.492)	(0.039, 0.197)
*z*		−7.168	−7.161	−7.079
*P*		<0.001	<0.001	<0.001

TIA, trabecular-iris angle at 500 *μ*m; AOD500 (the angle open distance at 500 *μ*m) was defined as the distance between a point on the trabecular network at 500 *μ*m from the scleral spur, a point where a line perpendicular to the posterior cornea surface intersects with the anterior iris surface. TISA500 (the trabecular-iris space area at 500 *μ*m) was defined as the surface area of a trapezoidal region consisting of the four lines: AOD500; a line drawn from the scleral spur perpendicular to the trabecular meshwork toward the iris surface; the inner corneoscleral wall, and the anterior surface of the iris.

## Data Availability

The datasets used to support the findings of this study are available from the corresponding authors upon request.
